# Prevalence and healthcare seeking practice of work-related musculoskeletal disorders among informal sectors of hairdressers in Ethiopia, 2019: findings from a cross-sectional study

**DOI:** 10.1186/s12889-020-08888-y

**Published:** 2020-05-19

**Authors:** Tesfaye Hambisa Mekonnen, Getachew Guteta Kekeba, Jember Azanaw, Gebisa Guyasa Kabito

**Affiliations:** 1 Department of Environmental and Occupational Health and Safety, Institute of Public Health, College of Medicine and Health Sciences, University of Gondar, P.O. Box. 196, Gondar, Ethiopia; 2 Guno Bedele Zone Labor and Social Affairs Office, Oromia National Regional State, Oromia, Ethiopia

**Keywords:** Prevalence, Work-related musculoskeletal disorders, Hairdressers, Ethiopia

## Abstract

**Background:**

Work-related musculoskeletal disorders (MSDs) are the major threats to public health, with a significant impact on workers, employers, and the general population. Musculoskeletal disorder related to work not only results in adverse health effects such as physical injury, disability, and a reduction in workers’ quality of life, but it also places immense burdens on the use of healthcare facilities and a substantial loss of productivity. The purpose of this research was to discover the prevalence and associated factors of work-related MSDs among hairdressers in Oromia Special Zone Surrounding Finfine, Ethiopia.

**Methods:**

A cross-sectional survey was carried out between March and April 2019. We included a total of 699 hairdressers with a systematic random sampling technique. Work-related MSDs was evaluated with the standardized Nordic Musculoskeletal survey, and the survey was administered by the interviewer. We employed SPSS version 20 software to perform a bivariate and multivariate analysis. A *p*-value of < 0.05 was considered a significant association.

**Results:**

In total, 652 hairdressers were interviewed with a response rate of 93.2%. Participants’ mean age was 33.19 (SD ± 9.639) years. The prevalence of work-related MSDs was 70.2% (*N* = 458) [95% CI (66.7, 73.9)] and 55.7% in the past 12-months and 7 days, respectively. The study showed the highest prevalence rate was observed in shoulder 53.7% (*n* = 350) followed by 53.4% (*n* = 348) neck and 53.2% (*n* = 347) low back. Of the participants, 33.4% (*n* = 153) perceived their pain to be severe whereas 28% (*n* = 128) a high disabling. Almost one-third (*n* = 187) of the respondents used healthcare services. Age [AOR = 2.73; 95% CI (2.55, 5.46)], work experience [AOR = 1.51; 95% CI (1.03, 2.20)], number of customers served per day [AOR = 2.35; 95% CI (1.35, 4.11)], and hours spent standing to make hair [AOR = 3.4; 95% CI (2.49, 7.77)] were significantly associated factors.

**Conclusion:**

This study found work-related MSDs were prevalent among hairdressers, but the use of healthcare services remains low. Age, length of employment, number of customers served per day, and number of hours spent standing per day to make hair were significantly associated. Therefore, we recommend employers need to develop health and safety programs that account for factors related to the workplaces. The findings also demonstrate that health practitioners would encourage pain management procedures.

## Background

Musculoskeletal disorders (MSDs) are pains that mainly affect body structures such as tendons, muscles, joints, ligaments, nerves, and bones [1–3]. They are usually attributed to years of exposure to events in daily life (unrelated to the workplace) and to injuries caused by accidents at work [1, 4]. Musculoskeletal disorders induced by occupational exposures are the major threats to public health, with a significant effect on workers, employers, and the general population. The conditions contribute to considerable morbidities such as physical injuries and disabilities [2, 3, 5], a decrease in quality of life [5–9], and a substantial burden for the use of healthcare facilities [5, 7, 10]. Besides, according to the 2016 survey of Global Burden of Disease (GBD), musculoskeletal pains were the leading cause of disability-adjusted life years (DALYs) (61.6% of years lived with disabilities) [11].

Studies have shown that musculoskeletal disorders related to work are commonly reported and widely documented in various professions, resulting in a considerable loss of productivity [1, 12, 13]. According to the Health and Safety Executive (HSE) survey, in 2017/18, over 469,000 employees experienced work-related musculoskeletal injuries in the UK, accounting for 35% of all work-related illnesses [1]. Informal/small scale industries in developing countries, including Ethiopia are growing enormously. However, the observance of health and safety regulations are usually neglected, and work environments are arranged to a substandard which make the situation more complicated [13, 14]. The hairdressing industry is one where the majority of the working population is likely subject to various physical, chemical (hair products), ergonomic, psychosocial, and biological hazards, concomitantly [14–19].

Specific ergonomic hazards such as extended standing, awkward work postures, strenuous and excessive shoulder movements, forceful exertion, and repetitive motion are inherently linked to hairdressing tasks [14, 20–22]. As a result, scholars indicated musculoskeletal pain among hairdressers is recurrently reported. For instance, a study conducted among Taiwanese hairdressers showed the prevalence of pain in shoulder was 91.7, 83.3% low back, and 75% neck [23]. Similarly, a study in Nigeria reported the prevalence of 76.3, 62.5, and 46.3% in low back, shoulder, and neck, respectively [4]. In our previous studies, we found 55.7% prevalence of back pain, 39.4% knee/ leg, 25.6% ankle [24], and 56.7% upper body [19].

Evidence unveils that the incidence of work-related musculoskeletal disorders is influenced by numerous factors. Accordingly, work-related musculoskeletal pain is notably attributed to socio-demographic characteristics such as gender [3, 25], age [19, 26–28], marital status [27], level of education and experience [29] and income [30]. Moreover, psychosocial characteristics such as work dissatisfaction [31], perceived work stress [32], and time pressure [3] are important factors of work-related musculoskeletal disorders. Intensive studies have established that occupational/workplace factors such as working hours [16], job tenure [3], work-demand [33], working posture [4, 16, 19], number of customers serviced per day [4], and shiftwork [27, 33] are the main contributors of work-related musculoskeletal pains. Further, lifestyle behaviors such as physical exercise, alcohol use, and smoking [19] also affect the occurrence of occupational related MSDs significantly.

In Ethiopia, there has been a significant rise in informal industries including hairdressing salons since recent decades [19]. However, the implementation of health and safety regulations is generally fragile. Besides, inspection personnel also usually find it difficult to get access to the industries for regular supervision purposes because of the lack of reliable data available [34]. To date, very limited studies have been conducted in these industries which provide little data for policy conclusions. Therefore, this study aimed to examine the prevalence and associated factors of musculoskeletal disorders, and the use of healthcare services among informal workers in hairdressing salons in Oromia Special Zone Surrounding Finfine, Ethiopia.

## Methods

### Study design

A cross-sectional

### Study period and area

This study was executed between March and April 2019 at Oromia Special Zone Surrounding Finfine, Ethiopia. The zone is one of the zones of the National Regional State of Oromia, and it was established in 2008 G.C. The zone surrounds Addis Ababa, also known as ‘Finfinne’ in the local language of ‘Afan Oromo.’ Addis Ababa is the capital of Ethiopia. The city is the administrative hub of the zone. The zone contains ten towns, namely Burayu, Dukam, Galan, Holeta, LagaTafo-LagaDadi, Sebeta, SebetaHawas, Menagesha, Sandafa, and Sululta. Among these, Burayu, Sebeta, Sebeta Hawas, and Sululta were selected by a lottery method for inclusion. According to the 2007 survey of the Central Statistics Agency (CSA) of Ethiopia, the total population of the zone was 794,489 of whom 228,420 were urban residents [35]. There were 2066 hairdressing salons in the zone with 2-4workers at each in the salon during the data collection period.

### Source and study populations

All hairdressers in Oromia Special Zone Surrounding Finfine were the source population whereas the randomly selected hairdressers from the included towns were the study population. Hairdressers who worked at least 12 months before the start of the study were eligible for inclusion, whereas those who had a history of injuries, musculoskeletal disorders, and pregnant women were excluded because they might confound the results.

### Sample size determination

OpenEpi software has been used to calculate the required sample, taking a prevalence of 75.6% [4], an absolute precision of 4% with a CI of 95% at Z = 1.96 critical value and a design effect of 1.5. A 10% was added for no responses that yielded the final sample size of 699. We also estimated sample for factors affecting work-related MSDs to ensure the sample is adequate. We performed this using a study in Brazil (80% proportion with 2.78 odds ratio for not comfortable body posture) [14], and we attained the sample size of 508. Comparing the two sample sizes, a decision has been made on the largest sample already estimated; 699. We employed a systematic random sampling technique to get the participants that were sampled. All the towns were first considered (included in chance) for a random selection, but only four were chosen by chance (lottery method). Because of the feasibility concerns, we included the towns with a lottery method.

### Operational definitions

#### Work related musculoskeletal disorders

Is a self-report pain, ache or discomfort in at least two body sites in the past 12 months. The body sites include neck, shoulder, upper back, lower back, hips /thigh, knee/leg, ankle/foot, elbow and wrist/hand [36].

#### Perceived disability

A pain disability point score of 3–6 points [37].

#### Perceived severity

A pain intensity score of ≥50 or < 3 disability points [37].

#### Awkward postures (AP)

Working with the neck bent without support, working with a bent wrist, working with the back bent without support, squatting and kneeling for two or more hours per day [38].

#### Repetitive work (RW)

A work involving repeating the same motion with less than 30 s or no variation every few seconds for two or more hours per day [38].

#### Static postures (SP)

Sitting or standing in a restricted space for two or more hours without changing positions per day [38].

#### Job satisfaction

A generic job satisfaction scales core of 32 or above [39].

#### Job stress

A workplace stress scale score of 21 or above [40].

#### Body mass index

Underweight = < 18.50; Normal =18.50–24.99, and Overweight/Obese = ≥ 25.00 [19].

#### Physical exercise

Performing any kind of sport activity at least two times per week for a duration of 30 min [41].

#### Adjustable chair

Chairs have wheels or castors suitable for the floor surface, have adjustable seat height [42].

#### Un-comfortable thermal environment

At least two or more “yes” responses in the thermal comfort factor items [43].

### Data collection tools and procedures

A structured, close-ended, and a pretested questionnaire was used for data collection via a face-to-face interview-administered technique. We developed the questionnaire after a meticulous review of literature with a slight modification [19, 37, 39, 40, 44]. The standardized Nordic musculoskeletal questionnaire was used for the assessment of musculoskeletal symptoms [44]. We analyzed the perceived satisfaction of hairdressers with the generic job satisfaction questionnaire on the 10-item scale [39]. The perceived job-related stress of the participants was collected with the workplace stress questionnaire on the 8-item scale [40]. The perceived severity and disability of musculoskeletal pains have also been evaluated by use of the 7-items Von korff et al. [37]. This instrument is measured based on 0 to 10 responses and added together to geta summary score of 100. The perceived severity of pain was calculated by the formula = (((response question 1) + (response question 2) + (response question 3)) / 3) * 10; disability score = (((response question 5) + (response question 6) + (response question 7)) / 3) * 10 and disability points = (points for disability days or question number 4) + (points for disability score). A final score was categorized into two with a score of ≤50 or disability point < 3 = 0 (low severity), a score of ≥50 or disability points < 3 = 1 (high severity) and perceived disability = disability points < 3 = 0 (low disability) and disability points ≥ 3 = 1 (high disability). All the instruments used in the current study have been employed in the previous studies conducted in the country’s context [19, 45]. Detail information on socio-demographic characteristics such as sex, age, religion, educational status, marital status, monthly salary, and work experience were gathered. A workplace-related factors including working hours per day, health and safety training, number of customers per day, work posture, and rest break were also explored. Finally, data on psychosocial factors such the perceived job satisfaction and job stress and behavioral/ individual factors including physical exercise, alcohol use, smoking, and body mass index (BMI) (self-report weight divided by height squared), and history of systemic illnesses were obtained.

### Data quality control

First, we focused on developing the instruments for data collection. As such, the questionnaire was designed in English and translated into local the language ‘Afan Oromo’ and back to English by 2 independent language experts. Then we employed four trained data collectors (Occupational health and safety experts and Environmental health officers 2 from each) and two supervisors. Data collectors and supervisors were provided with adequate training and instructions on issues related to the clarity of the questionnaire, study aims, confidentiality of information and voluntary participation (consent) of the participants. The principal investigator supervised both the data collectors and supervisors. We also conducted a pretest on 5% (35 participants) of the sample in another town (Bishoftu) a week before the actual data collection. Based on the pretest, the number of questions and some words difficult to understand were modified.

### Data analysis

Data were entered to and cleaned in Epi-info version 7.2.1.0 and exported to Statistical Package for Social Science (SPSS) version 20 software for analysis. The descriptive findings were presented using percentages, frequency distribution and measures of central tendency. We verified the goodness of model fitness by use of Hosmer and Lemeshow (*p* = 0.391). The reliability of the standardized Nordic Musculoskeletal questionnaire was tested (Cronbach’s alpha = 90.93). The 10-items job satisfaction scale questionnaire was examined (Cronbach’s Alpha =0.723). We also examined the 8-items job stress scale questionnaire, and a Cronbach’s alpha result found 0.781. Reliability of the Von korff et al. item employed for the assessment of the perceived disability and severity of pains has also been evaluated (Cronbach’s alpha = 0.802). The instruments were, therefore, tolerable for their consistency in repeating what had previously been measured with those tools. A multicollinearity of the variables was evaluated by use of a collinearity check, which was analyzed using a variable inflection factor (VIF < 5). The associations between the dependent and independent variables were examined using a binary logistic regression analysis. Accordingly, explanatory variables with a *p*-value < 0.2 in the bivariable analysis were exported to the multivariable logistic regression model to further investigate effects of potential confounders. The significance level was obtained at ≤0.05 *p*-values with 95% CI. Adjusted odds ratios were also used to establish strength of the associations.

## Results

### Socio-demographic characteristics

A total of 652 hairdressers were interviewed with a response rate of 93.3%. More than half, 54.9% (*N* = 358) of the participants were males. Fifty-two percent (*N* = 339) of the respondents aged > 30 and ranged from 17 to 55 with the mean of 33.19 (Standard deviations (SD) ± 9.639) years. Regarding educational level, 73.8% (*N* = 481) attended a secondary school and above. Just lower than half, 47.5% (*N* = 310) were married **(**Table 1**).**

**Table 1:** Sociodemographic characteristics of hairdressers, Ethiopia, 2019 (*N* = 652)

Characteristic	Frequency (n)	Percent (%)
**Sex**		
Male	358	54.9
Female	294	45.1
**Marital status**		
Married	310	47.5
Single	256	39.3
Divorced	86	13.2
**Religion**		
Protestant	307	47.1
Orthodox	208	31.9
Muslim	103	15.8
Catholic	34	5.2
**Educational Level**		
Unable to read and write	10	1.5
Primary school	161	24.7
Secondary school and above	481	73.8
**Monthly salary**		
< 1800 ETB	385	59.1
1800–2350 ETB	122	18.7
> 2350 ETB	145	22.2
**Work experience**		
≤ 5 years	342	52.5
> 5 years	310	47.5
**Age**		
≤ 30 years	313	48
> 30 years	339	52

Keys: *ETB* Ethiopian birr (currency), *N* Number, *WMSDs* Work-related musculoskeletal disorders

### Individual (behavioral) characteristics

Seventy-three percent (*N* = 476) of the respondents did not use alcohol, whereas 17.8% (*N* = 116) did. Eighty percent (*N* = 524) of the respondents were non-smokers, 6.4% (*N* = 42) past smokers, and 13.2% (*N* = 86) current smokers. About 46 % (*N* = 298) performed physical exercise of whom 6.3% (*N* = 41) performed one time, 19.2% (*N* = 125) 2 times, and 20.2% (*N* = 132) ≥ 3 times per week for at least 30 min. Twenty nine percent (*N* = 190) indicated they had a systemic illness. About 66.6% (*N* = 305) of the participants perceived low severity pain while 33.4% (*N* = 153) high. More than two third, 72.1% (*N* = 330) perceived a low disability pain whereas 27.9% (*N* = 128) perceived high.

### Workplace characteristics

Fifty-six percent (*N* = 365) of the participants worked more than 8 h per day. Lower than half, 48% (*N* = 313) took safety trainings. Few, 3.7% (*N* = 24) took more than a rest break of 45-min a day except for a lunch break. The majority, 53.2% (*N* = 347) of the respondents worked 7 days per week whereas 45.2% (*N* = 295) 4–6 days. Around 39.3% (*N* = 256) of the hairdressers spent 6–10 h per day standing at work to make hair. About more than half, 55.2% (*N* = 360) worked in the same position. In response to the question; the number of customers served in a day, 36.2% (*N* = 236) indicated that they served lower than 9 customers while 27.9% (*N* = 182) 10–15 customers per day (Table 2).

**Table 2:** Workplace characteristics of hairdressers, Ethiopia, 2019 (*N* = 652)

Characteristic	Frequency (n)	Percent (%)
**Awkward posture/ bending or twisting/ involved**		
Yes	314	48.2
No	338	51.8
**Hour spent bending/twisting per day**		
1–5 h	206	31.6
≥ 6 h	107	16.4
**Work in the same position/static posture**		
Yes	360	55.2
No	292	44.8
**Involved in repetitive task**		
Yes	451	69.2
No	201	30.8
**Working hours per day**		
≤ 8 h	287	44
> 8 h	365	56
**Working days per week**		
1–3 days	10	1.5
4–6 days	295	45.3
7 days	347	53.2
**Average rest break used per day**		
< 30 min	579	88.8
30–45 min	49	7.5
> 45 min	24	3.7
**Adequate light at work**		
Yes	554	85
No	98	15
**Work load**		
Yes	374	57.4
No	278	42.6
**Job categories /activities**		
**Washing and cutting**		
Yes	424	95.1
No	22	4.9
**Designing**		
Yes	219	33.6
No	433	66.4
**Rolling/curling/waving**		
Yes	197	30.2
No	455	69.8
**Coloring**		
Yes	19	2.9
No	633	97.1
**Adjustable chair available**		
Yes	327	50.2
No	325	49.8
**Finger supported scissors**		
Yes	465	71.3
No	187	28.7
**Thermal comfort**		
Comfortable	48	7.4
Uncomfortable	604	92.6
**Safety/ergonomic training**		
Yes	313	48
No	339	52
**Number of customers served per day**		
≤ 9 customers	198	30.4
10–15 customers	166	25.5
16–23 customers	130	19.9
≥ 24 customers	158	24.2
**Hours spent in standing position per day**		
1–5	269	41.3
6–10	230	35.3
11–15	153	23.4

### Psychosocial characteristics

The majority, 68.9% (*N* = 449) of the participants had a good work relationship with their colleagues and 64.9% (*N* = 423) with their customer. Almost all, 85.6% (*N* = 558) had boss at their work of whom 47.1% (*N* = 307) had a good work relationship with them. More than half, 55.7% (*N* = 363) were not satisfied with their current job. Moreover, 48 % (*N* = 315) explained they perceived stress because of their jobs.

### Prevalence of work-related musculoskeletal disorders and its health service use

**Fig. 1:**
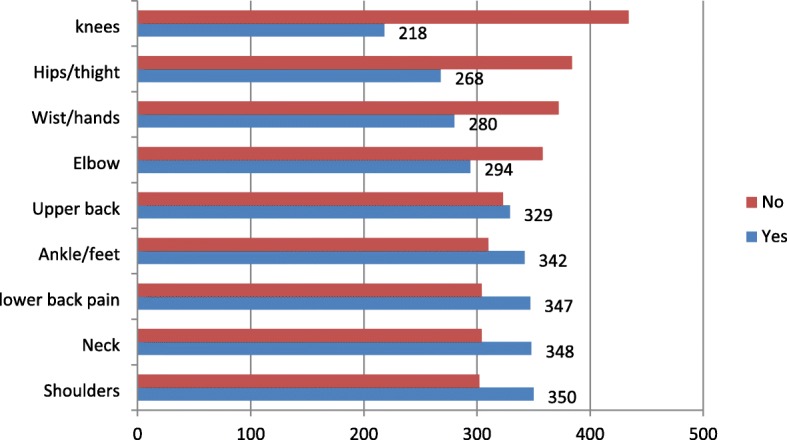
Prevalence of work-related MSDs by body regions

This study revealed that the overall prevalence of musculoskeletal disorder in the past 12 months at least in two body sites was 70.2% (*N* = 458) [95% CI (66.7, 73.9)] and the previous 7 days prevalence was 55.7% (*N* = 364) [95% CI (52, 59.4)]. A few, 28.7% (*n* = 187) of those participants with pains used healthcare services. The body region mostly sited with the symptoms was shoulder, 53.7% (*n* = 350) followed by neck, 53.4% (*n* = 348), and 53.2% (*n* = 347) low back while 33.4% (*n* = 218) reported in knee, 41.1% (*n* = 268) hips/thighs, and 42.9% (*n* = 280) wrists/hands (Fig. 1). Of the total respondents with shoulder pain, 45.4% (*n* = 159) experienced it in the last 7 days, 30% (*N* = 105) were prevented from their activities and 17.0% (*N* = 59) visited physician. About 32% (*N* = 111) of those with neck pain were prevented from their work, 16.1% (*n* = 56) visited physicians in the past 12 months and 31.4% (*N* = 109) had experienced it in the last 7 days. Moreover, among the sufferers of elbows/forearms pain, 38.7% (*N* = 114) experienced it in the past7 days, whereas 38% (*N* = 164) were prevented from their normal activities and 20.7% (*N* = 61) visited physicians.

### Factors associated with work-related musculoskeletal disorders

In the bivariable logistic regression analysis, age, physical exercise, educational level, work experience, number of customers in a day and hours spent standing to make hair were associated factors of work-related musculoskeletal disorders. In the multivariable logistic regression model, age, work experiences, number of customers in a day, and hours spent standing to make hair remained significantly associated with work-related musculoskeletal disorders.

The study identified age of the respondents was significantly associated with work-musculoskeletal disorders. The participants in the age group of > 30 years had 2.7 times higher odds of developing work-related MSDs than those in the age group of ≤30 years [AOR: 2.73; 95% CI (2.55, 5.46)]. Those who had a work experience of > 5 years were 1.5 times higher at risk to develop work-related musculoskeletal disorders compared with those with ≤5 years [AOR: 1.51; 95% CI (1.03, 2.20)].

The chance of developing work-related musculoskeletal disorders was 2.4 times higher for the participants who served > 24 customers a day than ≤9 customers [AOR: 2.4; 95% CI (1.35,4.11)]. The participants who spent 11–15 h per day standing to make hair were 3.4 times more likely to experience work-related musculoskeletal disorders than who spent 1–5 h [AOR: 3.4; 95% CI, (2.49,7.77)] (Table 3).

**Table 3:** Factors associated with work-related MSDs among hairdressers, Ethiopia, 2019

Characteristic (*N* = 652)	Work-related MSDs		COR (95% CI)	AOR (95% CI)	*P*-value
	Yes (%)	No (%)			
**Age**					
≤ 30 years	179(57.2)	134(42.8)	1	1	
> 30 years	279(82.3)	60(17.7)	3.48(2.43–4.98)	2.73(2.55–5.46)	0.0001*
**Educational level**					
Unable to read and write	9(81.2)	1(18.2)	4.12(0.52–32.79)	2.19(0.23–20.99)	0.49
Primary school	119(75.6)	42(24.4)	1.29(0.87–1.94)	1.06(0.68–1.64)	0.81
Secondary and Above	330(67.8)	151(32.2)	1	1	
**Work experience**					
≤ 5 years	217(63.5)	125(36.5)	1	1	
> 5 years	241(77.7)	69(22.3)	2.01(1.42–2.85)	1.51(1.03–2.20)	0.001*
**Physical exercise per week**					
Once per week	33(80.5)	8(19.5)	1	1	
2 times per week	91(72.8)	34(27.2)	0.65(0.27–1.54)	0.41(0.28–2.29)	0.69
≥ 3 times per week	84(63.6)	48(36.4)	0.42(0.18–0.99)	0.37(0.17–1.31)	0.15
**Hours spent in standing position per day**					
1–5 h	170(63.2)	99(36.8)	1	1	
6–10 h	154(66.9)	76(33.1)	1.18(0.82–1.71)	1.12(0.75–1.68)	0.94
11–15 h	134(87.6)	19(12.4)	4.11(2.39–7.05)	3.4(2.49–7.77)	0.0001*
**Customer attending per a day**					
≤ 9 customers	151(63.9)	85(36.1)	1	1	
10–15 customers	122(67)	60(33)	1.15(0.76–1.72)	1.14(0.73–1.78)	0.57
16–23 customers	74(75.5)	24(24.5)	1.74(1.02–2.95)	1.52(0.07–3.45)	0.06
≥ 24 customers	111(81.6)	25(18.4)	2.49(1.50–4.16)	2.35(1.35–4.11)	0.001*

Keys: -*ignificant in multivariate analysis; *N* Number, *OR* Odds ratio, *CI* Confidence interval, *WMSDs* Work-related musculoskeletal disorders

## Discussion

So far, occupational-related health conditions of informal workers have been least investigated in Ethiopia. The purpose of this study was to investigate the prevalence and associated factors of work-related musculoskeletal disorders among hairdressers in Oromia Special Zone Surrounding Finfine, Ethiopia. The study also described experiences of the workers on the use of healthcare for pain management. The finding showed that the prevalence of work-related MSDs among hairdressers in the past 12 months was 70.2% [95% CI (66.7, 73.9)] whereas 55.7% prevalence was reported during the past 7 days. This implies that work-related musculoskeletal disorders are the priority occupational health problems among informal sectors of hairdressers in Ethiopia. This may be because in Ethiopia, employees of small-scale industries including hairdressers and self-employed workers (as the majority of hairdressers are self-employed) are often rarely considered in health and safety policy actions and plans. Few recently introduced (as promising regulations) on health and safety issues in the country are also at the earliest stage and informal sectors are often ignored, despite their unremitting growth in number [46].

The prevalence observed in the current study was equivalent to the result in Brazil (71%) [14]. The inherent nature of work characteristics in informal industries is perhaps similar in every country, irrespective of the efforts to protect workers’ health and safety. However, our finding indicates a lower prevalence of musculoskeletal symptoms than a study finding in Nigeria (75.6%) [4]. The possible explanations could be because of discrepancies in workplace illness and injury reporting cultures and procedures, pain perceptions of workers and number of customers served daily.

Our investigation demonstrates that 53.4% of the participants reported pain in necks, which was comparable to data in Nigeria (46.3%) [4], Brazil (47%) [14], and Iran (50.9%) [47] but higher than another study in Ethiopia (29.3%) [19]. The current study also revealed that the prevalence of work-related musculoskeletal pain found highest in shoulder, 53.7% (*n* = 350) than in the other body sites. This is consistent with the data in Brazil (49%) [14]. The methods of data collection used in the studies were similar which might be the possible reason for this correspondence. However, a lower prevalence rate was detected in the current study than a report in Nigeria (62.5%) [4] and Taiwan (83.3%) [23]. The incidence of upper back pain was reported in 50.5% of the interviewed individuals, which is far higher than the Nigeria (4.6%) [4] and Iran (39.5%) [22] reports. Lower than half (45.1%) of the participants indicated having suffered from pain in their elbows which was far different from literature in Iran (17.6%). The probable suggestions for the two discrepancies (upper back and elbow) could be because of sample size, data collection technique, perceived severity of pains and injury reporting cultures. We also noticed that 42.9% of the respondents in the current study located pain in wrists/hands, which was higher than another report in Ethiopia (32.4%) [19], the United Kingdom (UK) (25%) [48] and Iran (15.1%) [22]. The inconsistent finding could be as a result of sample size and data collection technique.

Self-report pain in low back was indicated in 53.2% of the participants in the current investigation. The literature in Nigeria (76.6%) [4] and Iran (58.7%) [22] documented higher prevalence than our result did whereas a report in the UK registered a lower prevalence rate (27%) [48]. Almost half, 52.5% of hairdressers have also sited pain in knee, which is higher than a study in Iran (41.3%) and Nigeria (32.8%) [4].

In our analysis, a significant relation was observed between age of participants and work-related musculoskeletal disorders. This result is supported by findings from the others studies [19, 28, 49]. This might be because the biological structures of human body, particularly those related to bone and muscles would tend to degenerate as age increases. Consequently, functional capacity of the connective tissues and muscle strength would tend to decrease as age increases. This is also evident that as age increases, the likely occurrence of musculoskeletal pains would possibly increase. This explanation agrees with the studies in Greek [50] and the UK [1].

In this study, length of employment has been markedly associated with the development of work-related musculoskeletal pain. The result is congruent to a study conducted in Ethiopia [19], Pakistan [29], Nigeria [51], and Brazil [14]. This could be speculated that pain development might be related to the cumulative effects of workloads on the musculoskeletal structures when employment duration increases. A study conducted in Finland, however, contradicts this finding [52]. The study argued pains in the musculoskeletal systems likely increase for the newly employed workers (might be because of deficient in adaptation to work environment) than for the experienced ones. Variations in study designs used and the target populations involved in the studies seem to vary those findings. But, the extent to which work acclimatization is applicable to reduce work exposures related to ergonomic hazards remains unclear in that study.

A significant association of hours spent standing and the prevalence of musculoskeletal disorders was detected in the current investigation. This result concords the reports in Malaysia [53] and Brazil [4]. These similarities could be explained in part by that the extended standing might exert stress on the skeletal structures because of the possible increasing pressures on them. A prolonged standing might also bring about muscle stiffness, which in turn would result in impaired muscle functions. A previous survey has also presented a corresponding explanation [19].

The number of customers served in a day was the other significant factors of work-related musculoskeletal disorders. Our result corroborates the report in Nigeria [4]. The increased number of customers served per day would likely increase workload which in turn prohibits workers from utilizing the required rest breaks. Moreover, workers in small scale industries are usually limited in number/work alone, and the lack of support is often prominent which has also been correspondingly explained by a study in Greece [54].

This study plays a vital role to employers and other stakeholders because it assists them to formulate health and safety programs in informal sectors. It also inspires other investigators to further study the relations of a range of workplace factors and the development of pains in musculoskeletal structures. However, due to the relatively large sample size included, we have not performed a posture analysis that could address the degree to which those participants have been exposed to the workplace ergonomic factors. Moreover, because the study used a self-report assessment method, a recall bias has not been ruled out, resulting in under estimation of the result.

## Conclusions

The study found that work-related MSDs were prevalent among hairdressers but the use of healthcare services continues to be low. Age, length of employment, number of customers served per day, and number of hours spent standing per day were significant factors. Therefore, we advise employers need to develop health and safety programs that account for factors relevant to the workplaces. The findings also demonstrate that health practitioners would encourage pain management procedures.

## Data Availability

Authors presented the data in the main paper and made available from the corresponding author on a reasonable request.

## Abbreviations

AOR: Adjusted Odd Ratio
BMI: Body Mass Index
CI: Confidence Interval
COR: Crude Odd Ratio
ETB: Ethiopia Birr
MSDs: Musculoskeletal Disorders
SD: standard deviations
USA: the United States of America.
SPSS: Statistical Package for Social Science
WMSDs: Work-related Musculoskeletal Disorders
